# Multi-Information Flow CNN and Attribute-Aided Reranking for Person Reidentification

**DOI:** 10.1155/2019/7028107

**Published:** 2019-02-06

**Authors:** Haifeng Sang, Chuanzheng Wang, Dakuo He, Qing Liu

**Affiliations:** ^1^School of Information Science and Engineering, Shenyang University of Technology, Shenyang, Liaoning 110870, China; ^2^College of Information Science and Engineering, Northeastern University, Shenyang, Liaoning 110004, China

## Abstract

This paper presents a multi-information flow convolutional neural network (MiF-CNN) model for person reidentification (re-id). It contains several specific multilayer convolutional structures, where the input and output of a convolutional layer are concatenated together on channel dimension. With this idea, layers of model can go deeper and feature maps can be reused by each subsequent layer. Inspired by an image caption, a person attribute recognition network is proposed based on long-short-term memory network and attention mechanism. By fusing identification results of MiF-CNN and attribute recognition, this paper introduces the attribute-aided reranking algorithm to improve the accuracy of person re-id further. Experiments on VIPeR, CUHK01, and Market1501 datasets verify the proposed MiF-CNN can be trained sufficiently with small-scale datasets and obtain outstanding accuracy of person re-id. Contrast experiments also confirm the availability of the attribute-assisted reranking algorithm.

## 1. Introduction

Person reidentification (re-id) refers to matching and recognizing the identities of pedestrians captured by multicameras with nonoverlapping views, which is significant to improve the efficiency of the security system. Owing to the low resolution of cameras, it is hard to obtain discriminative face features, so the current person re-id methods are mainly based on visual features of pedestrians, such as color and texture [1]. In practice, changes in viewpoint, pose, and illumination among different camera views, as well as partial occlusions and background clutters, pose a great challenge to person re-id [2].

Two principal person re-id methods are feature representation and metric learning. Feature representation seeks to find features with stronger discrimination and better robustness to represent pedestrians. Many kinds of features have been utilized for this, in which appearance features are the simplest and the most popular ones. Color, texture, and shape are the features that can be extracted for human appearance [3]in feature representation, such as HSV color histogram, LBP texture, and Gabor features, and then used for reidentifying people with similarity among pedestrian features. Attribute features are also widely used in person re-id. Common attributes include gender, length of hair, and clothing. These attributes are highly intuitive and understandable descriptors which have proved to be successful in several tasks, such as face recognition and activity recognition [4]. Although attribute features are complicated in terms of extraction and expression, they contain rich semantic information and are more robust to illumination and viewpoint changes. Therefore, the combination of attribute features and low-level features can effectively improve the accuracy of person re-id [5]. The metric learning methods employ the machine learning algorithm to learn a good similarity metric, which makes the feature similarity of the same pedestrian greater than that of different pedestrians.

In recent years, deep learning has shown great success in a variety of tasks in image classification and frequency domain [6], where CNN is particularly outstanding. Compared with the traditional methods, CNN has stronger feature learning ability, and the learned features are more intrinsically representative to the original data, so it has better performance in extracting image features. Two types of CNN models are commonly employed in the community. The first type is the classification model as used in image classification and the second is the Siamese model using image pairs or triplets as input [7]. Most of the existing public datasets of person re-id only contain thousands of pedestrian image samples; a small number of training samples can easily lead to overfitting, which limits the performance of person re-id model. In addition, the deep neural networks for person re-id are similar in structure; that is, the feature maps extracted by convolutional layer are directly fed into the next convolutional layer [8–12]. Such structure usually ignores the correlation among features of each layer, thus reducing the mobility of feature information to some extent. In the process of back propagation, as the number of layers in neural network deepens, the gradient update information may attenuate in exponential form and cause vanishing gradient problem.

This work proposes to develop a modified deep neural network model for person re-id that could reduce overfitting caused by the lack of training samples. Moreover, this work aims to improve the identify accuracy of person re-id network with assistance of pedestrian attribute recognition. To this end, contribution of this paper is three-fold: first, this paper designs a multi-information flow convolutional neural network (MiF-CNN) to solve the person re-id problem. The network contains a series of multi-information flow convolution structures which connect the input and output of each convolutional layer together, realizes the reuse of features, and enhances the feature information flow and gradient back propagation of the entire network. Second, this paper designs a person attribute recognition network (PARN) based on long-short-term memory (LSTM) network and attention mechanism. The PARN decodes pedestrian visual features extracted by MiF-CNN into attribute features and outputs the attribute words of each person. Third, this paper presents an attribute-aided reranking algorithm which rematches attribute features among samples to aid more positive samples rank higher in rank list so as to improve the identify accuracy further.

The rest of this paper is organized as follows.  Section 2 reviews the state of the art for person re-id.  Section 3 introduces the details of MiF-CNN.  Section 4 shows the principle of the PARN. The proposed attribute-aided reranking algorithm is detailed in Section 5. The experimental results and analysis are given in Section 6. Finally, conclusion and future works are discussed in Section 7.

## 2. Related Works

The early person re-id methods extract the manually designed features to represent pedestrians. Farenzena et al. divided pedestrian images into multiple areas and extracted three complementary kinds of features, weighted color histograms, maximally stable color regions, and recurrent high-structured patches. Then, match these features and measure the similarity between pedestrian pair [13]. Yang et al. proposed a novel salient color names based color descriptor (SCNCD), which was utilized to guarantee that a higher probability will be assigned to the color name near to the color. Based on SCNCD, color distributions in different color spaces were fused into feature representation for person [14]. Bazzani et al. proposed asymmetry-based HPE descriptor, which accumulated HSV histogram of multiple pedestrian images as a global appearance feature and detected patches portraying highly informative recurrent ingredient in local regions as local feature [15]. Wu et al. designed a novel gradient self-similarity (GSS) feature based on HOG to capture the patterns of pairwise similarities of local gradient patches. The combination of HOG and GSS achieved improvement in person re-id accuracy [16].

Apart from manually designed low-level features, attribute features that represent mid-level semantic information apply to person re-id as well. Compared with low-level descriptors, attributes are more robust to image translations [7]. Layne et al. labeled 15 binary attributes for the VIPeR dataset and trained SVM to detect attributes. They also learned a weighted L2-norm distance metric to fix each attribute and fused them with low-level visual features [17]. Wang et al. predicted complete attribute vector by exploiting both visual feature and marked attributes and obtained the overall ranking list by fusing the rank result from visual features and attribute vectors separately [18]. Chen et al. learned attribute of person by part-specific CNN and merged them with another identification CNN embedding in a triplet structure for person re-id task [19]. Wang et al. proposed a deep neural network that contains an auto-encoder model to learn hidden attributes of person from visual feature in an unsupervised manner, which alleviated the requirement of massive annotation [20].

Deep learning has become popular for solving person re-id problems in recent years. Ahmed et al. present a deep CNN architecture for person re-id. The architecture computed differences in feature values across the two views around a neighborhood of each feature location to add robustness to positional differences in corresponding features of the two input images [12]. Cheng et al. proposed a novel multichannel CNN. After the first layer of CNN, features were divided into four equal parts that aimed to learn features for the respective body part. The proposed CNN was trained with improved triplet loss function [21]. Lin et al. used ResNet [22] as the base network to learn low-level features and attributes jointly, and trained network with combining the person re-ID loss and attribute prediction loss [23]. Yan et al. proposed an attention block which learned par-level attention on different local regions, and integrated the proposed block into existing CNN structures for training with the identify loss [24]. Inspired by above works, this paper proposes a multi-information flow convolutional neural network to extract discriminative pedestrian features. In addition, this paper designs a person attribute recognition network based on LSTM and attention mechanism for improving person re-id results with assistance of attributes recognition.

## 3. Multi-Information Flow Convolutional Neural Network

**Figure 1:**
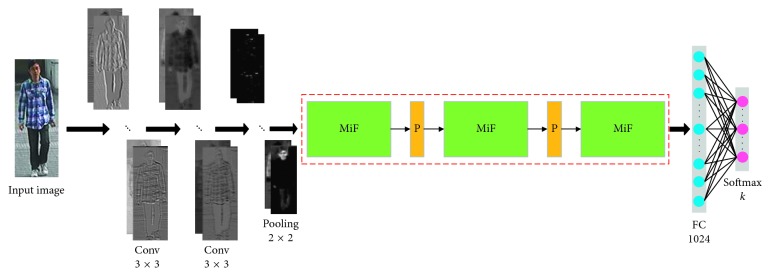
Architecture for MiF-CNN, where green rectangle denotes the multi-information flow convolutional structures, orange rectangle denotes the middle pooling layer, and *k* is the number of pedestrian categories in the training set.

The proposed MiF-CNN solves the person re-id problem with classification thought. The overall network structure is shown in Figure 1. The structure includes 2 shallow convolutional layers, 3 multi-information flow convolutional structures with novel connection pattern, fully connected layers, max pooling layers, and classification output layers. Low-level features of pedestrian images are extracted first by 2 shallow convolutional layers. After deeper multi-information flow convolutional structures, MiF-CNN extracted higher level features. The final discriminative pedestrian feature vectors are obtained after reducing dimensions by pooling layers and integrating by fully connected layers.

### 3.1. Features Extraction

In MiF-CNN, all convolutional filters are 3 × 3 with stride 1. Batch normalization and ReLU activation function are applied after each convolutional layer. The operation process of convolutional layer can be formulated as

(1) $$\begin{array}{c} \begin{array}{cc} \left\{\begin{array}{c} z_{j}^{\left(l\right)}=\underset{i}{\sum }x_{i}^{\left(l-1\right)}\;⊗\;w_{j}^{\left(l\right)},\\ x_{j}^{\left(l\right)}=\sigma \left(z_{j}^{l}\right), \end{array}\right. & l>1 \end{array}, \end{array}$$

where *x*_*i*_^(*l* − 1)^ is the *i*‐th feature map from the (*l* − 1)‐th convolutional layer, *z*_*j*_^(*l*)^ is the convolutional output of *x*_*i*_^(*l* − 1)^, *x*_*j*_^(*l*)^ is the *j*‐th feature map from the *l*‐th convolutional layer, *w*_*j*_^(*l*)^ is the filter on the *j*‐th feature map in the *l*‐th convolutional layer, and ⊗ represents the convolutional operation. The process of convolutional layers extracting features is that neuron on the *j*-th feature map in the *l*‐th convolutional layer sum each feature map after connecting and convolution by filter *w*_*j*_^(*l*)^, and map the extracted features on *j*‐th feature map in the *l*‐th convolutional layer. *σ*(·) is the ReLU activation function, which is formulated as *σ*(*x*)=max(0, *x*). Because of batch normalization, bias is ignored.

### 3.2. Multi-Information Flow Convolutional Structure

**Figure 2:**
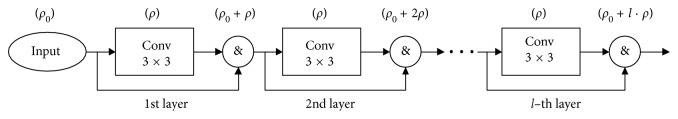
Multi-information flow convolutional structures, where “&” represents concatenate operation on channel dimension.

In this structure, both output and input of the current convolutional layer are concatenated together and fed into the next convolutional layer; i.e., the input of each layer is the connection combination of outputs from all previous layers. The detail of multi-information flow convolutional structures is shown in Figure 2.

This connection pattern makes feature maps of each layers be reused by all subsequent layers in forward propagation process, which makes the whole CNN model learn more feature information of pedestrian images. It can be considered as a special “Data Augmentation” in feature maps so as to enhance the information mobility of the network. In back propagation process, gradient of input in each layer contains derivative of loss function with respect to input, which makes propagation of gradient more effective and network easier to be trained. In multi-information flow convolutional structure, the number of feature maps that each layer outputs is a constant value *ρ*, so the number of feature maps that *l*‐th layers outputs is *ρ*_0_+*ρ*(*l* − 1), where *ρ*_0_ is the number of feature maps in the initial layer. Supposing the feature map of *l*‐th channel in the initial layer is *x*_*i*_^(0)^, where *i* ∈ (1, *ρ*_0_), then the feature map of *j*‐th channel that initial layer outputs can be expressed as

(2) $$\begin{array}{c} z_{j}^{\left(1\right)}=\underset{i}{\sum }x_{i}^{\left(0\right)}\;⊗\;w_{j}^{\left(1\right)}, \end{array}$$

where *w*_*j*_^(1)^ is the weight of the initial layer. The output after activation function is

(3) $$\begin{array}{c} a_{j}^{\left(1\right)}=\sigma \left(z_{j}^{\left(1\right)}\right), \end{array}$$

where *j* ∈ (1, *ρ*). The feature map that the *l*‐th layer outputs can be expressed as

(4) $$\begin{array}{c} \begin{array}{cc} \left\{\begin{array}{c} z_{q}^{\left(l\right)}=\underset{p}{\sum }x_{p}^{\left(l-1\right)}\;⊗\;w_{q}^{\left(l\right)},\\ a_{q}^{\left(l\right)}=\sigma \left(z_{q}^{\left(l\right)}\right), \end{array}\right. & l>0, \end{array} \end{array}$$

where *p* ∈ (1, *ρ*_0_+*ρ*(*l* − 1)), *q* ∈ (1, *ρ*). The output of the *l*‐th layer after concatenate operation is

(5) $$\begin{array}{c} x_{r}^{\left(l\right)}=\left[x_{p}^{\left(l-1\right)},\;\;a_{q}^{\left(l\right)}\right],\;l>0, \end{array}$$

where *r* ∈ (1, *ρ*_0_+*ρ* · *l*), [·, ·] represents the concatenate operation on channel dimension.

In the process of back propagation, supposing Δ*x*_*r*_^(*l*)^ is the derivative of loss function with respect to *x*_*r*_^(*l*)^. Due to *x*_*r*_^(*l*)^ containing *x*_*p*_^(*l* − 1)^ and *a*_*q*_^(*l*)^, it produces two parts of gradient as shown below:

(6) $$\begin{array}{c} \left\{\begin{array}{c} \Delta a_{q}^{\left(l\right)}=\Delta x_{r}^{\left(l\right)}\cdot \frac{\partial x_{r}^{\left(l\right)}}{\partial a_{q}^{\left(l\right)}},\\ \Delta x_{p}^{\left(l-1\right)}=\Delta x_{r}^{\left(l\right)}\cdot \frac{\partial x_{r}^{\left(l\right)}}{\partial x_{p}^{\left(l-1\right)}}, \end{array}\right. \end{array}$$

where Δ*a*_*q*_^(*l*)^ is the gradient of output from the *l*‐th layer after activation function. Δ*x*_*p*_^(*l* − 1)^ is the gradient of output from the (*l* − 1)‐th layer. The gradient of weight in the *l*‐th layer is

(7) $$\begin{array}{c} \left\{\begin{array}{c} \Delta z_{q}^{\left(l\right)}=\Delta a_{q}^{\left(l\right)}\cdot \frac{\partial a_{q}^{\left(l\right)}}{\partial z_{q}^{\left(l\right)}}=\Delta x_{r}^{\left(l\right)}\cdot \frac{\partial x_{r}^{\left(l\right)}}{\partial a_{q}^{\left(l\right)}}\cdot \sigma^{\prime }\left(z_{q}^{\left(l\right)}\right),\\ \Delta w_{q}^{\left(l\right)}=\Delta z_{q}^{\left(l\right)}\cdot \frac{\partial z_{q}^{\left(l\right)}}{\partial w_{q}^{\left(l\right)}}=\Delta z_{q}^{\left(l\right)}\cdot x_{p}^{\left(l-1\right)}, \end{array}\right. \end{array}$$

where Δ*z*_*q*_^(*l*)^ is the gradient of the convolution result in the *l*‐th layer, *σ*′(*z*_*q*_^(*l*)^) is the derivative of the activation function with respect to *z*_*q*_^(*l*)^, and Δ*w*_*q*_^(*l*)^ is the gradient of weights in the *l*‐th layer. The network utilizes Δ*w*_*q*_^(*l*)^ to update weights of each layer, which is formulated as

(8) $$\begin{array}{c} {\left(w_{q}^{\left(l\right)}\right)}_{\text{new}}=w_{q}^{\left(l\right)}-\eta \cdot \Delta w_{q}^{\left(l\right)}, \end{array}$$

where *η* is the learning rate. Gradient keeps back propagation to the (*l* − 1)‐th layer. The gradient of *x*_*p*_^(*l* − 1)^ is

(9) $$\begin{array}{c} \Delta x_{p}^{\left(l-1\right)}=\Delta z_{q}^{\left(l\right)}\cdot \frac{\partial z_{q}^{\left(l\right)}}{\partial x_{p}^{\left(l-1\right)}}=\Delta z_{q}^{\left(l\right)}\cdot w_{q}^{\left(l\right)}. \end{array}$$

As shown in equations (6) and (9), the loss function produces two flows of gradient with respect to outputs of each convolutional layer, which makes error information propagating more effective in network and restrains the vanishing gradient to a certain extent.

The proposed MiF-CNN includes three multi-information flow convolutional structures. Between every two multi-information flow convolutional structures, a middle pooling layer is applied to compares the redundancy features. Hyperparameter *ρ* is set with a small value. When it comes to a new multi-information flow convolutional structure, *ρ* is doubled. Such a design makes each convolution layer learn a small quantity of features and reduce the redundant features so as to optimize the efficiency of network. With deeper layers, the network can learn more high-level and complex pedestrian features and improve the final identification accuracy.

### 3.3. Loss Function

Current deep learning algorithms usually use cross-entropy loss as cost function, which is formulated as

(10) $$\begin{array}{c} L_{\text{S}}=-\frac{1}{M}\overset{M}{\underset{i=1}{\sum }}y^{\left(i\right)}\;\log\frac{e^{\theta_{y\left(i\right)}^{T}x^{\left(i\right)}}}{\sum_{j=1}^{k}e^{\theta_{j}^{T}x^{\left(i\right)}}}, \end{array}$$

where *y*^(*i*)^ is the ground truth of pedestrian categories in training set, *θ* is the parameter of the last fully connected layer, *x*^(*i*)^ is the feature vector of training samples, *k* is the number of pedestrian categories in the training set, and *M* is the batch size.

However, in practice, when using cross-entropy loss merely, if the quality of extracted features is not good enough, it will lead to intraclass distance being greater than interclass distance. Aiming at this problem, Wen et al. proposed center loss in 2016 [25]. Combination of cross-entropy loss and center loss can enhance the discrimination and generalization ability of the network. Center loss is defined as follows:

(11) $$\begin{array}{c} L_{\text{C}}=\frac{1}{2M}\overset{M}{\underset{i=1}{\sum }}{\left\|x_{i}-c_{j}\right\|}_{2}^{2}, \end{array}$$

where *c*_*j*_ is the center of the *j*‐th pedestrian feature and *x*_*i*_ is the feature vector of pedestrian. Center loss minimizes the distance between feature and its center in order to reduce the intraclass distance. Center *c*_*j*_ is updated with equation (12):

(12) $$\begin{array}{c} \left\{\begin{array}{c} \Delta c_{j}^{t}=\frac{\sum_{i=1}^{M}\delta \left(y_{i}=j\right)\cdot \left(c_{j}^{t}-x_{i}\right)}{1+\sum_{i=1}^{M}\delta \left(y_{i}=j\right)},\\ c_{j}^{t+1}=c_{j}^{t}-\beta \cdot \Delta c_{j}^{t}, \end{array}\right. \end{array}$$

where *β* is the update rate, *δ*(*y*_*i*_=*j*) is 1 if prediction equals to ground truth, otherwise is 0. That is to say, center is updated only when network predicts correctly.

## 4. Person Attributes Recognition Network

**Figure 3:**
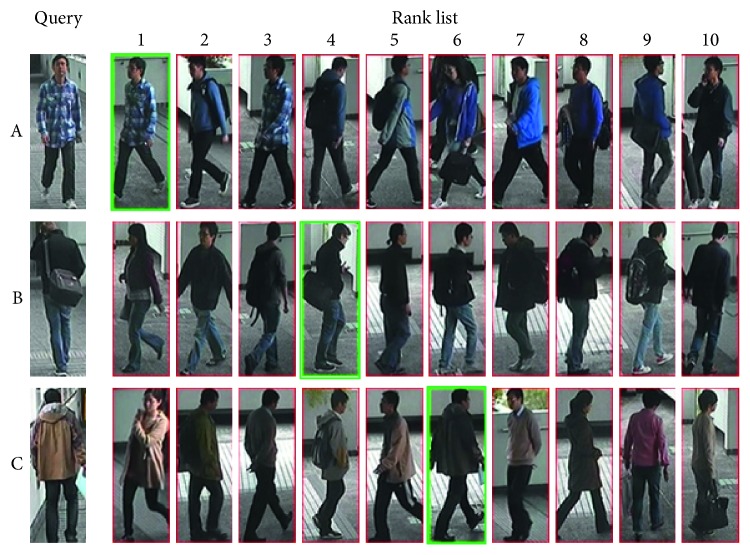
Identification results of MiF-CNN (green bounding box represents positive samples in gallery).

The rank list of MiF-CNN is shown in Figure 3. In the incorrect identification results of rank 1 (pedestrian B and pedestrian C), there is a big difference in attribute features between the top-ranking negative samples and the query image, including gender, clothing, whether carrying handbag or not, and so on. Hence, this paper recognizes person attributes for improving accuracy of person re-id.

**Figure 4:**
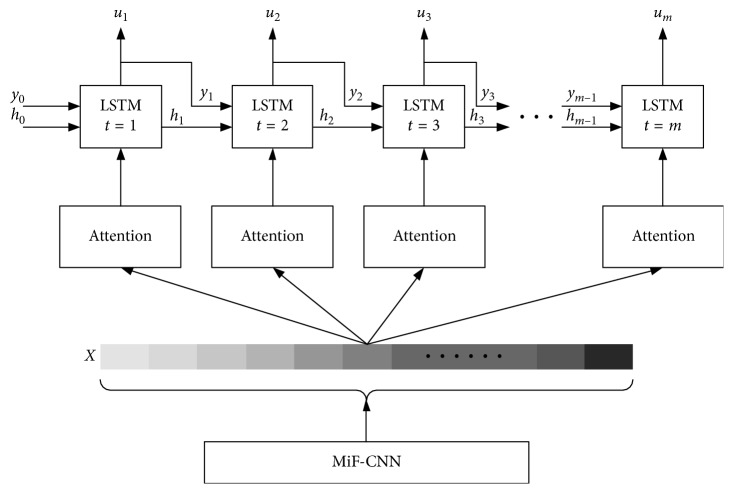
Architecture of PARN.

Based on Encoder-Decoder idea, Xu et al. [26] proposed a neural network model that can learn and generate the content of images. The model utilized CNN as encoder to extract features of images which were then fed into a recurrent neural network (RNN) for decoding into language captions of images. Inspired by that, this paper presents a person attribute recognition network (PARN) with LSTM and attention mechanism. The proposed PARN takes pedestrian features that are extracted by MiF-CNN as input and outputs the attributes information of pedestrian images. The architecture of PARN is demonstrated in Figure 4.

### 4.1. Input of PARN

In PARN, the input is the feature maps that are before the last fully connected layer in the MiF-CNN. The input feature is split into *n* feature vectors, each of which corresponds to a part of the image. Each feature vector is a *D*_*X*_-dimensional vector which is represented as

(13) $$\begin{array}{c} X=\left\{x_{1},x_{2},...,x_{n}\right\},\;x_{i}\in {\mathbb{R}}^{D_{X}}. \end{array}$$

Referring to the natural language processing method, PARN transforms words in person attribute labels into word embedding. As a part of the input of PARN, each word embedding is a *D*_*Y*_-dimensional vector:

(14) $$\begin{array}{c} y=\left\{y_{1},y_{2},...,y_{m}\right\},\;y_{i}\in {\mathbb{R}}^{D_{Y}}, \end{array}$$

where *m* is the number of attribute words in each pedestrian image and *y*_*i*_ is the word embedding corresponding to each attribute word.

For attribute words, common one-hot encoding considers each word as an individual, which ignores the correlation among words. However, word embedding represents each word as a continuous dense vector, which makes those correlative words closer in space.

### 4.2. Attention Mechanism

Attention mechanism has been widely used in natural language processing and computer vision. By measuring the correlation between the output and different parts of the input, attention mechanism gives different weights to different parts of the input, enabling the network to use more important feature information for prediction and reduce the dimension of input data [27].

**Figure 5:**
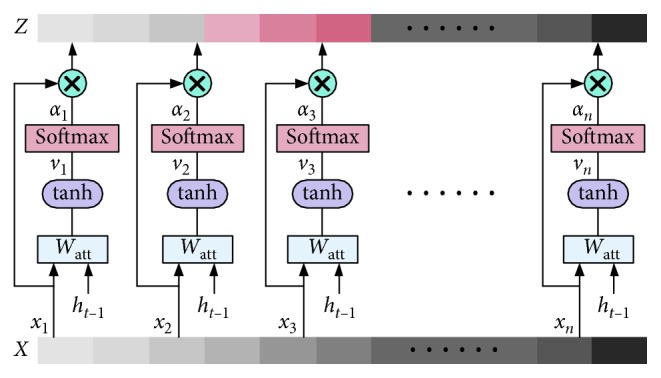
Schematic diagram of attention mechanism.

In practice, a certain attribute of pedestrian is only corresponding to a certain part of the image. For example, when recognizing whether a pedestrian is wearing a hat, people only pay attention to the area above the head of the pedestrian usually, instead of other areas irrelevant to the attribute. Therefore, before feeding the image features into the LSTM network, attention mechanism is introduced to calculate the correlation between different positions of image features and the hidden state of LSTM at the previous time. The schematic diagram of attention mechanism is shown in Figure 5.

At time *t*, the fully connected layer and tanh function are used to integrate the information of the input feature vector and the hidden state of LSTM at the previous time, which is formulated as

(15) $$\begin{array}{c} v_{i}=\tanh\left(W_{\text{att}X}x_{i}+W_{\text{att}h}h_{t-1}\right), \end{array}$$

where *W*_att*X*_ and *W*_att*h*_ represent the fully connected layer weights of *x*_*i*_ and *h*_*t*−1_, respectively. The attention weights *α*_*i*_ is obtained by supplying softmax function on score vector *v*_*i*_:

(16) $$\begin{array}{c} \alpha_{i}=\text{softmax}\left(W_{\text{att}v}v_{i}\right), \end{array}$$

where *W*_att*v*_ represents the fully connected layer weights of *v*_*i*_. The output *Z* is the weighted sums of all *α*_*i*_:

(17) $$\begin{array}{c} Z=\overset{n}{\underset{i=1}{\sum }}\alpha_{i}x_{i}. \end{array}$$

Feature *Z* highlights the local features that are helpful to predict and suppress the other local features that make small contribution to prediction, which reduces the dimension of features to some extent and makes LSTM network focus on the part of input features that have greater correlation with prediction while recognizing person attributes so that improves the efficiency and prediction accuracy of the network.

### 4.3. LSTM

**Figure 6:**
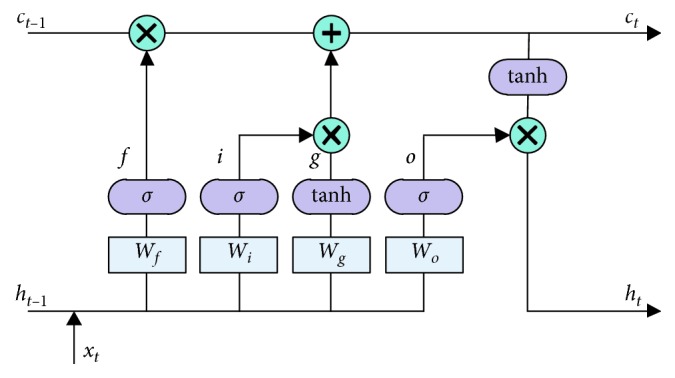
Architecture of the LSTM unit.

Normal RNN updates network parameters with back propagation, which easily suffers from the vanishing gradient when there is a long gap between relevant information and the current position to predict. LSTM network solves this problem well because of its own structure advantage. The architecture of LSTM unit is shown in Figure 6.

Here, *c* is the cell state for storing and transferring information, *f* is the forget gate which decides what should be abandoned in the cell state, *i* is the input gate which decides what should be stored in the cell state, *g* represents a vector of new candidate values that should be added to the cell state, *o* is the output gate which decides what parts of the cell state should be output to the next time, *h* is the hidden state of LSTM, *x* is the input of LSTM, and *t* denotes the current time.

In PARN, at any time *t*, the input of LSTM consists of two parts: word embedding *y*_*t*−1_ at the previous time and image features *Z*_*t*_ at the current time. The operation processing of LSTM in PARN is formulated as

(18) $$\begin{array}{c} \left\{\begin{array}{c} f=\text{sigmoid}\left(W_{f}\left[h_{t-1},y_{t-1},Z_{t}\right]+b_{f}\right),\\ i=\text{sigmoid}\left(W_{i}\left[h_{t-1},y_{t-1},Z_{t}\right]+b_{i}\right),\\ g=\text{sigmoid}\left(W_{g}\left[h_{t-1},y_{t-1},Z_{t}\right]+b_{g}\right),\\ o=\text{sigmoid}\left(W_{o}\left[h_{t-1},y_{t-1},Z_{t}\right]+b_{o}\right),\\ c_{t}=f\;⊙\;c_{t-1}+i\;⊙\;g,\\ h_{t}=o\;⊙\;\tanh\left(c_{t}\right), \end{array}\right. \end{array}$$

where *W* and *b* represent the weights and biases of each gate, respectively. Sigmoid function outputs a number between 0 and 1 to decide the quantity scale of values that go through these gates. Tanh function is the activation function of input. This design makes LSTM give different weights for information at different times so that it can choose what part of information to remember or forget in a long-term sequence.

The time step of LSTM in PARN is set as the number of attributes of each pedestrian image *m*. The output hidden state *h*_*t*_ is calculated by a fully connected layer and softmax function at each time step to predict pedestrian attribute words.

It is worth noting that, at each time step, the input word embedding of LSTM is the one that LSTM learned at the previous time step, which takes advantage of the LSTM when solving the long-term sequence problem. Because of the correlation among attributes of pedestrian, like the pedestrian owning attribute “*male*” generally own attribute “*short hair*” and attribute “*pants*,” LSTM can utilize the previous attribute result to predict the current attribute more accurately.

### 4.4. Network Optimization

The loss function of PARN includes two parts, one is the cross-entropy between prediction and ground truth of network, which is expressed as

(19) $$\begin{array}{c} L_{\text{sa}}=\overset{m}{\underset{i=1}{\sum }}u^{\prime }\;\log\left(\frac{e^{\theta_{ai}^{T}u}}{\sum_{j=1}^{n_{w}}e^{\theta_{aj}^{T}u}}\right), \end{array}$$

where *u* is the prediction value of the network, *u*′ is the ground truth of pedestrian labels, *m* is the time step of LSTM, and *n*_*w*_ is the total number of attribute words in each dataset. As shown in equation (19), the loss of a single image in single epoch is the cumulated loss value after *m* iterations. The other one is the loss function of attention mechanism as described in [40]:

(20) $$\begin{array}{c} L_{\alpha }=\frac{1}{2n}{\overset{n}{\underset{i=1}{\sum }}\left(1-\overset{m}{\underset{i=1}{\sum }}\alpha_{i}^{j}\right)}^{2}, \end{array}$$

where *α*_*i*_^*j*^ represents the attention weights of the image feature *x*_*i*_ and *n* is the number of parts in the image feature. The object function of PARN to optimize is

(21) $$\begin{array}{c} J=-\frac{1}{M}\overset{M}{\underset{i=1}{\sum }}\left(L_{sa}^{i}+\lambda \cdot L_{\alpha }^{i}\right), \end{array}$$

where *M* is the batch size and *λ* is the rate of *L*_*α*_ in the object function.

## 5. Attribute-Aided Reranking Algorithm

Given a probe image *q*_0_ and gallery image set *G*={*g*_1_, *g*_2_, ..., *g*_*N*_} including N pedestrian images, the initial similarity distance between *q*_0_ and *g*_*i*_ is computed with the Euclidean distance as

(22) $$\begin{array}{c} d\left(q_{0},g_{i}\right)=\sqrt{\overset{N}{\underset{i=1}{\sum }}{\left(x_{q_{0}}-x_{g_{i}}\right)}^{2}}, \end{array}$$

where *x*_*q*_0__ and *x*_*g*_*i*__ represent the features of *q*_0_ and *g*_*i*_, respectively, that extracted by MiF-CNN. The initial rank list *R*_0_={*g*_1_^0′^, *g*_2_^0′^, ..., *g*_*N*_^0′^} is obtained by sorting similarity distance *d*(*q*_0_, *g*_*i*_) in ascending order, where *d*(*q*_0_, *g*_*i*_^0′^) < *d*(*q*_0_, *g*_*i*+1_^0′^). If the top-1 gallery *g*_1_^0′^ is the positive sample, it means rank 1 is correct. If the positive sample is not the top-1 gallery but the top-5 gallery, it means rank 5 is correct.

The attribute-aided reranking algorithm reranks the rank list *R*_0_ according to the attribute feature similarity between *q*_0_ and *g*_*i*_ so that more positive gallery gets higher ranking in *R*_0_, which could improve the performance of person re-id. To be specific, when rank 5 is correct but rank 1 is not, the proposed algorithm distributes the initial feature score *s*_f_ for top-5 gallery *g*_1_^0′^ ~ *g*_5_^0′^ according to their ranking in *R*_0_, the higher the ranking, the higher the *s*_f_. Then, the proposed algorithm distributes attribute score *s*_a_ for *g*_1_^0′^ ~ *g*_5_^0′^ according to the number of their attributes that are the same as *q*_0_, the more same attributes, the higher *s*_a_. The total score of each gallery is *s*=*s*_f_(1 − *γ*)+*s*_a_*γ*, where *γ* is the score weight. The reranking rank list *R*_0_^*∗*^ is obtained by reranking *g*_1_^0′^ ~ *g*_5_^0′^ in descending order of their total score *s*. For *M* query images, the whole processing of the attribute-aided reranking algorithm is shown as Algorithm 1.

## 6. Experiments

This paper evaluates the attribute recognition accuracy of PARN on person re-id public datasets first and then compares our results with other person attribute recognition methods. Then, this paper evaluates the performance of MiF-CNN on three people re-id datasets and the availability of the proposed attribute-aided reranking algorithm for improving the accuracy of person re-id. The analysis of experiment results is also given after comparing our results with the state-of-the-art person re-id methods.

### 6.1. Datasets and Evaluation Protocol

This paper evaluates the proposed methods on three challenging person re-id public datasets including VIPeR [28], CUHK01 [29], and Market1501 [30].

 
*VIPeR Dataset*. It contains 1264 images of 632 persons. Because of the lack of image samples, low-resolution, a great change in pose, view, and illumination, it is one of the hardest difficult person re-id datasets. In experiment, the VIPeR dataset is randomly split into two parts: images of 316 persons for training and the images of remaining 316 persons for testing.
 
*CUHK01 Dataset*. It consists of 3884 images of 971 persons which are captured by two cameras with different views. 485 pedestrians are randomly chosen for training and other 486 pedestrians for testing.
 
*Market1501 Dataset*. It is one of the largest person re-id datasets that include 32268 images of 1501 persons. Each person has a number of images that are captured by six cameras with different views. The dataset is divided into two parts: 12936 images of 751 persons for training and 19732 images of 750 persons for testing.
 
*Evaluation Protocol*. For the single-shot datasets VIPeR and CUHK01, cumulative match characteristic (CMC) is used to record the ranks of correct identify [31]. For the multishot dataset Market1501, apart from CMC, mean Average Precision (mAP) is utilized to evaluate the performance of the proposed methods.

### 6.2. Experimental Results on Attribute Recognition

**Figure 7:**
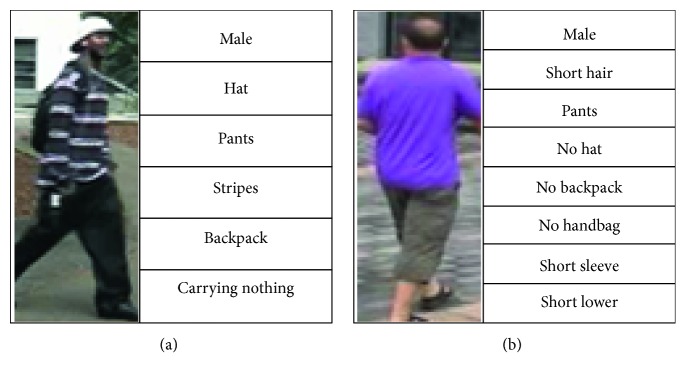
Attribute label of the pedestrian in datasets.

The attributes in PARN refer to pedestrian identity-level attributes. For the VIPeR dataset, attributes include “*gender*,” “*length of hair*,” “*lower clothing*,” “*upper clothing*,” “*backpack or not*,” and “*carrying anything or not*.” For the CUHK01 dataset, attributes include “*gender*,” “*length of hair*,” “*backpack or not*,” “*handbag or not*,” “*color of upper*,” and “*color of lower*.” For the Market1501 dataset, attributes contain “*gender*,” “*length of hair*,” “*wearing hat or not*,” “*lower clothing*,” “*backpack or not*,” “*handbag or not*,” “*length of sleeve*,” and “*length of lower clothing*.” Each person in each dataset owns an attribute word list as shown in Figure 7.

This paper evaluates the attribute recognition accuracy of PARN on the Market1501 dataset and compares our results with outstanding person attribute recognition method APR [22]. The experiment results are reported in Table 1, where “*L.slv*” represents the “*length of sleeve*” and “*L.low*” represents the “*length of lower clothing*.”

It can be observed from Table 1 that PARN obtains superior recognition accuracy of 8 attributes, especially “length of hair,” “handbag or not,” and mean accuracy are higher than APR, whereas accuracy of other attributes is closer with APR. Comparison with APR demonstrates the outstanding attribute recognition performance of PARN which plays an important role in improving the accuracy of person re-id.

### 6.3. Experimental Results on Person re-id

This paper evaluates the performance of the proposed methods on three datasets. The experimental results of the proposed methods and other state-of-the-art methods are presented in Table 2.

As shown in Table 2, the proposed MiF-CNN model obtains a great performance among state-of-the-art methods, and on the contrary, comparison between MiF and MiF + PARN demonstrates that the proposed attribute-aided reranking algorithm is helpful to increase the person re-id accuracy. The improvement effect is especially obvious on VIPeR and CUHK01 datasets, where the identification accuracy of rank 1, rank 5, and rank 10 improves by 6.02%, 6.33%, and 2.22% and 4.94%, 4.94%, and 2.26%, respectively. The proposed MiF-CNN model with the attribute-aided reranking algorithm gets the best rank 5 accuracy on the VIPeR dataset, the best rank 1 and rank 5 accuracy on the CUHK01 dataset, and the best mAP on the Market1501 dataset among various methods.

### 6.4. Analysis of Experimental Results

Because of the small quantity of training samples in VIPeR and CUHK01 datasets, a deep CNN model is hard to be trained and easily suffers from overfitting. To handle this, the FT-JSTL + DGD method based on deep CNN learned deep features from multiple domains jointly by merging all the datasets together and fine-tuned the pretrained model on VIPeR and CUHK01 separately. The structure and hyperparameters of the deep CNN model in the M3TCP method need to be adjusted manually for adapting the scale of different datasets so as to overcome the training problem of the deep neural network.

In contrast, the proposed MiF-CNN has an immobile structure and can be trained on smaller datasets directly without any fine tuning. In spite of the deep structure, the model can converge quickly and well. MiF-CNN without the attribute-aided reranking algorithm outperforms FT-JSTL + DGD and M3TCP by 0.27% and 13.17%, respectively, at rank 1 accuracy on the CUHK01 dataset. It also outperforms FT-JSTL + DGD by 2.22% at rank 1 accuracy on the VIPeR dataset, which indicates that the proposed MiF-CNN model has an excellent ability of reducing overfitting and generalization and an outstanding performance in person re-id.

**Figure 8:**
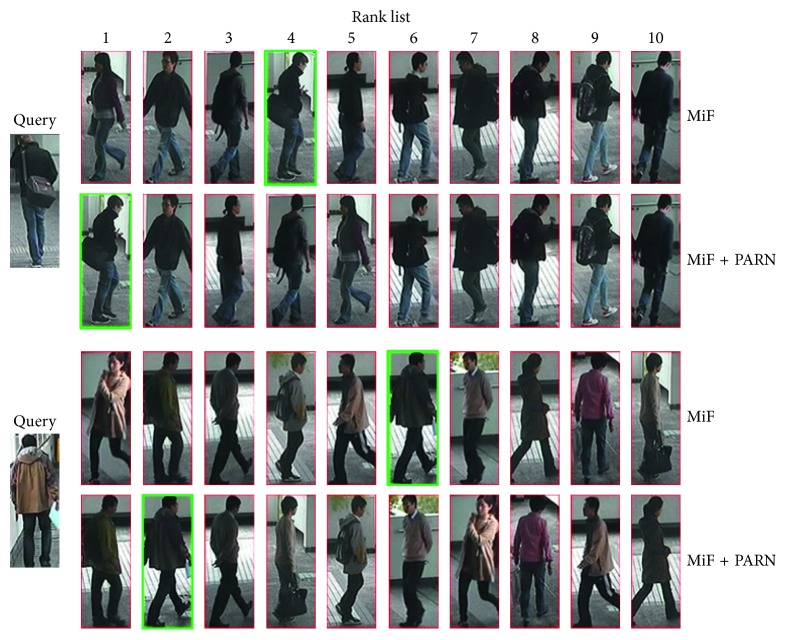
Rank list comparison between MiF and MiF + PARN.

Figure 8 demonstrates the rank list of MiF and MiF + PARN. It can be seen that the MiF-CNN with the attribute-aided reranking method enables the lower ranked positive sample in rank list of MiF-CNN obtain a higher ranking so as to improve the accuracy of person re-id, which verifies the effectiveness of the attribute-aided reranking algorithm for improving performance of person re-id model.

## 7. Conclusion

This paper studies and discusses the training problem of deep neural network in person re-id task, and the using of pedestrian attributes for further improving the accuracy of person re-id. The proposed MiF-CNN model realizes the reuse of feature maps and gradient information, which enhances the feature mobility of network and improves the efficiency of gradient propagation. The designed person attribute recognition network uses an attention mechanism to measure the correlation between input feature maps and the hidden state of LSTM at previous time for reducing the dimension of features. It also employs LSTM to decode image features into pedestrian attributes. On the basis of the attribute recognition results, the attribute-aided reranking algorithm is presented, which rematches attribute features among samples to aid more positive samples rank higher in rank list so as to improve the identify accuracy further.

The experimental results on three public person re-id datasets indicate the outstanding performance of MiF-CNN model in person re-id. The attribute-aided reranking algorithm makes a major contribution to improve the accuracy of person re-id. In the future, more improvement and optimization will be done on pedestrian attribute recognition network. Moreover, the caption of person images or videos can be obtained by the LSTM model with natural language process ideas which make person re-id methods more significant.

## Figures and Tables

**Algorithm 1:**
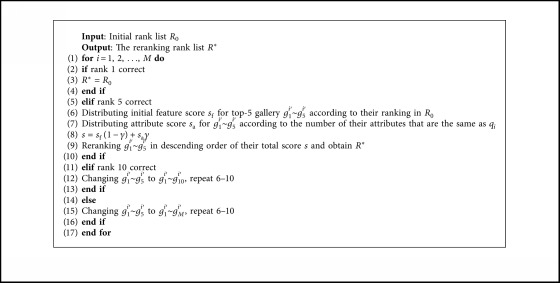
The attribute-aided reranking algorithm.

**Table 1:** Recognition accuracy of different attributes in PARN and APR (%).

Method	Gender	Hair	Clothes	Hat	Backpack	Handbag	L.slv	L.low	Mean
APR	85.78	83.46	91.36	88.21	86.32	76.01	94.12	92.64	87.23
PARN	84.93	79.10	89.35	96.67	83.86	85.18	92.84	88.45	87.55

**Table 2:** Comparison of various methods with the proposed methods on three datasets (%).

Methods	VIPeR			CUHK01			Market1501			
	Rank 1	Rank 5	Rank 10	Rank 1	Rank 5	Rank 10	Rank 1	Rank 5	Rank 10	mAP
DNS [32]	42.28	71.46	82.94	64.98	84.96	89.92	67.96	—	—	41.89
DLDA [33]	44.11	72.59	81.66	67.12	89.45	91.68	48.15	—	—	29.94
FT-JSTL + DGD [11]	38.6	—	—	66.60	—	—	—	—	—	—
PDC [34]	51.27	74.05	84.18	—	—	—	**84.14**	**92.73**	**94.92**	63.41
K-means-CNN [35]	46.50	69.30	80.70	53.50	82.50	91.20	—	—	—	—
Spindle [9]	**53.80**	74.10	83.20	—	—	—	76.90	91.50	94.60	—
PersonNet [36]	—	—	—	71.14	90.07	**95.00**	37.21	—	—	18.57
CSBT [37]	36.60	66.20	**88.30**	51.20	76.30	91.80	42.90	—	—	20.30
SDH-CNN [38]	—	—	—	—	—	—	58.12	68.50	80.82	48.20
M3TCP [21]	—	—	—	53.70	84.30	91.00	—	—	—	—
DM^3^ [39]	42.70	74.30	85.10	49.70	77.30	86.10	75.80	89.10	92.40	53.20
MiF	40.82	68.35	81.01	66.87	85.39	89.51	78.92	86.46	89.28	66.00
MiF + PARN	46.84	**74.68**	83.23	**71.81**	**90.33**	91.77	79.39	88.95	91.36	**66.46**

“MiF” represents the MiF-CNN method, and “MiF + PARN” represents the MiF-CNN with the attribute-aided reranking method.

## Data Availability

The (attributes recognition accuracy on the Market1501 dataset and person re-id accuracy on VIPeR, CUHK01, and Market1501 datasets) data used to support the findings of this study are included within the article.
